# Impact of the Mass Media on Adherence to the Mediterranean Diet, Psychological Well-Being and Physical Activity. Structural Equation Analysis

**DOI:** 10.3390/ijerph18073746

**Published:** 2021-04-03

**Authors:** Rafael Marfil-Carmona, Manuel Ortega-Caballero, Félix Zurita-Ortega, José Luis Ubago-Jiménez, Gabriel González-Valero, Pilar Puertas-Molero

**Affiliations:** 1 Faculty of Education Sciences of Granada, University of Granada, 18011 Granada, Spain; rmarfil@ugr.es (R.M.-C.); felixzo@ugr.es (F.Z.-O.); jlubago@ugr.es (J.L.U.-J.); pilarpuertas@correo.ugr.es (P.P.-M.); 2 Faculty of Education and Sport Sciences of Melilla, University of Granada, 52005 Melila, Spain; manorca@ugr.es

**Keywords:** physical activity, Mediterranean diet, media pressure, psychological well-being

## Abstract

Background: The influence of mass media on emotions, subjective well-being and behaviours in society should be clearly understood. Physical-health education has an important role to play as a preventive tool. The aim of this study was to develop an explanatory model regarding the relationships between mass media, psychological well-being, physical activity, Mediterranean diet and age and to compare the model with multi-group analysis according to gender. Methods: A descriptive, non-experimental, cross-sectional design was used, with 634 participants between 18 and 66 years old (M = 35.18 ± 9.68). Results: Structural equation modeling was found to be satisfactory for all parameters. Results show that mass media have a significant direct influence on well-being, with negative effects on physical activity and adherence to a Mediterranean diet. The model fitted better for males in terms of gender differences, showing a better fit of psychological well-being being associated with higher levels of physical activity and better adherence to the Mediterranean diet. Among women, no relationships were found between mass media pressure and psychological well-being and healthy physical habits, but higher personal satisfaction was associated with better physical activity and better dietary patterns. Conclusions: Thus, the study approaches society to a perspective influenced by mass media and physical-health education, reporting and emphasizing the importance of healthy lifestyles.

## 1. Introduction

In order to understand contemporary society, it is necessary to look at the influence of mass media on people, particularly in terms of aspects linked with the creation of values and behaviour patterns in younger populations [1]. Accordingly, authors such as Hong and Kim [2] and Wearn and Shepherd [3] found that audiovisual media have a persuasive effect on emotions, psychosocial aspects such as subjective wellbeing and social–behavioural patterns. Likewise, the educational context should take on great importance in the early stages of life in order to establish healthy physical habits and the responsible use of new technologies, as this has been shown to benefit individuals’ physical and mental health and quality of life [4,5,6,7,8]. In fact, when a habit or lifestyle is acquired in the early stages of education, it will rarely be forgotten in adulthood [9,10,11].

Specifically, mass media present a positive impression of iconic personalities through publicity, encouraging people to change their lifestyle, such as physical activity motivation or eating habits, thus promoting healthy habits [12,13]. However, not all mass media have a positive effect, as highlighted by Francisco et al. [14] and Stirling et al. [15], as excessive mass media exposure generates dissatisfaction, setting beauty standards and causing society’s demands to fit within established parameters.

Therefore, these factors may cause eating disorders, usually among younger women, who aim to respond to the standards fixed in their culture and society, including by excessive physical exercise. Thus, this leads individuals into an unhealthy lifestyle and reduces their emotional wellbeing [16,17]. Age and socio-economic factors are therefore a key issue in the adult population, as it has been shown that increasing age advocates a healthy state and improved quality of life, responsible use of mass media, consolidation of mental and emotional processes and improved psychological well-being [18,19,20,21,22].

Based on the above, it should be noted that low levels of physical activity and unhealthy behaviours among young adults and adolescents are a cause for concern and lead to significant socio-health problems [23]. In this sense, Meganto-Mateo et al. [24] showed that physical activity and an optimal diet are associated with greater psychological well-being, because these people perceive themselves as healthier, have lower levels of stress and have a better mood compared to people who do not engage in physical exercise. For the use and implementation of healthy physical activity, the guidelines proposed by the World Health Organization (WHO) [25], which state that around 27.5% of adults and 81% of adolescents do not comply with them, which causes serious problems in terms of non-communicable diseases and mental health, should be considered. Therefore, a balanced diet that provides all the nutrients necessary for the body should be adopted [26], with the Mediterranean diet being one of the best-known diets that meets all the requirements for healthy eating [27,28]. However, physical activity and adherence to the Mediterranean diet are associated with individual factors such as gender, age, socioeconomic status, educational level, living location and personality features [19,29].

In relation to the problems described above, in this study, a structural equation model is developed including physical activity and Mediterranean diet adherence as endogenous variables, taking into account the effect of mass media pressure, psychological well-being and age. Positive and negative relationships between the variables studied is found, highlighting the importance of lifelong health education. Furthermore, this association between variables can differ according to gender; thus, a multigroup analysis was carried out.

Based on the above, the following aims were set out: (a) to develop an explanatory model regarding associations between mass media, psychological well-being, physical activity, Mediterranean diet and age; (b) to contrast the structural model by multigroup analysis according to gender.

## 2. Materials and Methods

### 2.1. Subjects and Design

A descriptive, non-experimental (ex post facto) and cross-sectional design was used in this quantitative study. One single measurement was carried out for a single group using convenience sampling for participant selection. The study sample was composed of 634 participants, providing a homogeneous distribution by gender. Women accounted for 55.5% (*n* = 352) of the sample and men for 44.5% (*n* = 282). The age range of the participants was from 18 to 66 years (35.18 ± 9.68). Spanish subjects were invited to participate if they fulfilled the criteria of being over legal age and not exceeding ordinary retirement age. A total of 53 questionnaires were invalidated because they did not fulfil the inclusion criteria or because they were incorrectly completed.

### 2.2. Instruments and Variables

A self-reporting and self-completion questionnaire (ad-hoc) was used to compile socio-demographic information. Participants’ gender (categorized as male and female) and age were recorded. In addition, physical–sports aspects were recorded, showing how much time (expressed in minutes) subjects spent on physical activity or sport.

A questionnaire was used to assess social pressure regarding physical appearance. The original questionnaire “Sociocultural Attitudes Towards Appearance Questionnaire-4 (SATAQ-4)” was developed by Schaefer et al. [30] and translated into Spanish by Llorente et al. [31]. It is composed of 22 items using a Likert-type scale with five response alternatives (“1 = completely disagree” to “5 = completely agree”). The questionnaire assesses the dimensions of internalising thin/low body fat (items 3, 4, 5, 8 and 9); internalising an athletic/muscular build (items 1, 2, 6, 7 and 10), family pressure (items 11, 12, 13 and 14), peer pressure (items 15, 16, 17 and 18) and media pressure (items 19, 20, 21 and 22). In particular, the dimension of media pressure was used in this study, with an example of its items being “I feel pressure from the media to improve my appearance”. The internal reliability consistency in the present study was α = 0.916 across all items; more specifically, for media pressure, α = 0.967 was obtained.

In order to evaluate psychological well-being, a shortened version of the Psychological Well-Being Scale (PWBS) [32] was used, adapted to Spanish by Díaz et al. [33]. This scale is composed of 29 items answered on a Likert-type scale with six response options (“1 = totally disagree” to “6 = totally agree”), including 10 items that are inversely formulated. This instrument makes it possible obtain a summary of psychological well-being emerging from the following six dimensions of psychological well-being: self-acceptance (items 1, 7, 19 and 31), positive relationships (items 2, 8, 14, 26 and 32), autonomy (items 3, 4, 9, 15, 21 and 27), environmental mastery (items 5, 11, 16, 22 and 39), personal growth (items 24, 36, 37 and 38) and purpose in life (items 6, 12, 17, 18 and 23). The internal reliability of this scale in the present study for all items was α = 0.918.

The Mediterranean diet quality index (KIDMED) questionnaire developed by Serrá-Majem et al. [34] was used to measure adherence to the Mediterranean diet. This instrument is made up of 16 dichotomous items that represent Mediterranean diet standards. Four of the items are negatively rated (−1) if answered in the affirmative (e.g., “do you avoid breakfast?”). All other items are scored positively (+1) if the answer is yes (e.g., “do you regularly eat fresh or cooked vegetables/vegetables?”). An overall score ranging from −4 to 12 is obtained by summing the items, allowing the total of the scale to be obtained and to categorize adherence to the Mediterranean diet (low quality, ≤3; needs improvement, 4–7; optimal quality, ≥8).

### 2.3. Procedure

**Figure 1:**
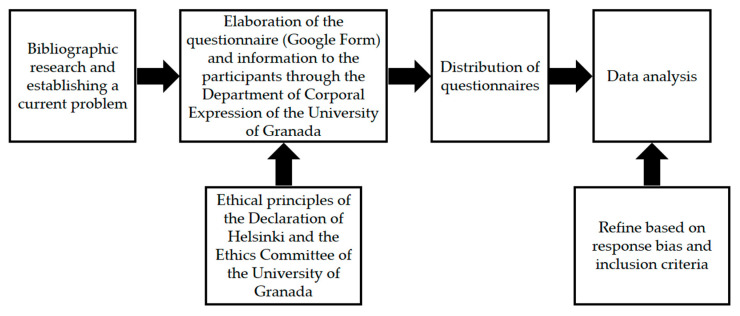
Study procedure.

As shown in Figure 1 of the study procedure, scientific research was carried out in order to gather information and clarify some of the current problematic situations in society. Afterwards, the Department of Didactics of Musical, Plastic and Corporal Expression of the University of Granada created a Google form with the specified instruments, describing the study aim, as well as accepting participation by sending the form. Several routes were used to administer the questionnaire, including social network dissemination. Two questions were duplicated in order to check that they had not been filled in randomly and to ensure the reliability of the answers. Therefore, the correct bias of the answers was ensured. Even so, 53 questionnaires were excluded because they did not comply with the inclusion criteria or were incorrectly completed. Research ethics principles established by the Declaration of Helsinki [35] were followed in this study, ensuring anonymity and respecting participants’ rights. In addition, research approval was granted by the Ethics Committee of the University of Granada (1230/CEIH/2020).

### 2.4. Data Analysis

**Figure 2:**
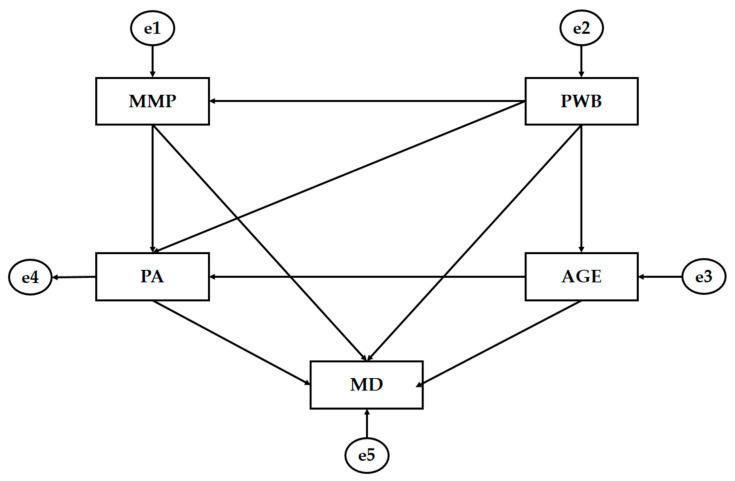
The theoretical model. Note: Mass media pressure (MMP); Mediterranean diet (MD); physical activity (PA); psychological well-being (PWB); age (AGE); error (e).

The statistical software SPSS 25.0 (IBM Corp, Armonk, NY, USA) was used to process the data in order to establish the frequencies and means of the basic descriptive analysis. The Cronbach’s coefficient was used to determine the internal consistency of the instruments, establishing the reliability index at 95%. Structural equation multigroup analysis (SEM) was performed with AMOS 23.0 software (IBM Corp., Armonk, NY, USA). SEM analysis was used to establish the relationships between the variables composing the theoretical model (Figure 2) for both groups (male and female). One general model was constructed for the study sample and two differentiated models in order to verify the relationships between variables according to the participants’ genders. The SEM developed for this analysis was based on five observable variables that provided an explanation for the relationships. For endogenous variables, causal explanations were made by considering the observed associations between indicators and measurement reliability. Therefore, the measurement error of the observable variables was included in the model and could be directly controlled and interpreted as multivariate regression coefficients. Unidirectional arrows represented influence lines between latent variables and were interpreted from regression weights. A significance level of 0.05 was established using Pearson’s Chi-square test.

The Mediterranean diet (MD) is an endogenous variable that is affected by mass media pressure (MMP), psychological well-being (PWB), physical activity (PA) and age. Thus, PA is affected by MMP, PWP and age, in the same manner as PWP affects MMP and age. Furthermore, in this case, they act as endogenous variables.

In order to verify the compatibility between the developed model and the empirical data obtained, the model fit was examined. Following the criteria proposed by Marsh [36], the reliability model was obtained according to the goodness-of-fit. For the Chi-square analysis, values associated with a non-significant *p*-value indicate a good model fit. Since this statistic is very sensitive to sample size effects, other fit indices should be used [37]. Additional parameters such as the comparative fit index (CFI), normalized fit index (NFI), incremental fit index (IFI) and the Tucker–Lewis index (TLI) were used. The values obtained needed to be be above 0.90 to represent an acceptable fit, and values above 0.95 represented an excellent fit. Moreover, the root mean squared error of approximation (RMSEA) was used, where an acceptable fit was determined by values of 0.08 and excellent fit with values below 0.05.

## 3. Results

The model developed through the evaluation of the variables assessed in an adult sample aged 18–66 years as a function of gender showed a good fit for all indices. The Chi-square analysis showed a significant *p*-value (X^2^ = 2.022; df = 1; pl = 0.155). However, these indicators could not be interpreted independently due to the influence of the sample size and susceptibility [36]. Therefore, other standardized fit indices were employed that were also less sensitive to the sample size.

The comparative fit index (CFI) analysis obtained a value of 0.995, which represented an excellent fit. The normalized fit index (NFI) analysis obtained a value of 0.991, the incremental fit index (IFI) was 0.995 and the Tucker–Lewis index (TLI) obtained a value of 0.953, all of which were excellent. The root mean square error of approximation analysis (RMSEA) also obtained an excellent value of 0.040.

**Figure 3:**
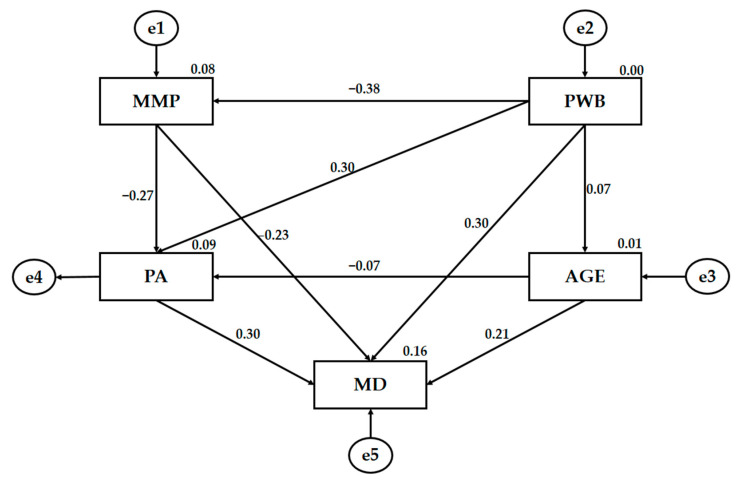
The structural equation for the theoretical model. Note: Mass media pressure (MMP); Mediterranean diet (MD); physical activity (PA); psychological well-being (PWB); age (AGE); error (e).

Figure 3 and Table 1 shows the regression weights of the theoretical model, with statistically significant relationships at *p* < 0.01 and *p* < 0.001. Mass media pressure (MMP) was negatively associated with psychological well-being (PWP) (*p* < 0.001; r = −0.379), physical activity (PA) (*p* < 0.001; r = −0.272) and Mediterranean diet adherence (MD) (*p* < 0.001; r = −0.235). The strength of the regression weights was medium–low for this case. However, PWB was positively related to PA (*p* < 0.001; r = 0.301) and MD (*p* < 0.001; r = 0.296), showing regression weights with a medium effect strength. In addition, MD was associated directly and positively with BP (*p* < 0.001; r = 302) and age (*p* < 0.01; r = 0.213), with a low–medium strength of the regression weights. No statistically significant differences were found between the existing relationship of age with BP and PWB.

The developed model according to male gender showed a good fit for all indexes. Chi-square analysis showed a significant *p*-value (X^2^ = 2.760; df = 1; pl = 0.097). Comparative fit index (CFI) analysis obtained a value of 0.993, the normalized fit index (NFI) obtained a value of 0.989 and the incremental fit index (IFI) was 0.993, representing an excellent fit. In the Tucker–Lewis index (TLI) analysis, a value of 0.927 was obtained, showing in this case an acceptable fit. The root mean square error of approximation analysis (RMSEA) obtained a value of 0.079, which is acceptable.

**Figure 4:**
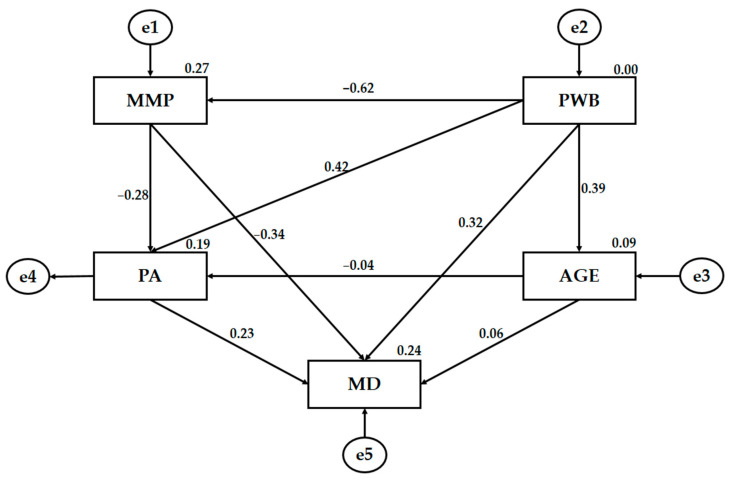
The structural equation for males. Note: Mass media pressure (MMP); Mediterranean diet (MD); physical activity (PA); psychological well-being (PWB); age (AGE); error (e).

Figure 4 and Table 2 show the regression weights of the male model, showing statistically significant relationships at levels *p* < 0.05, *p* < 0.01 and *p* < 0.001. The male model fitted better than the female model. MMP was negatively associated with PWP (*p* < 0.001; r = −0.618) in the case of a high strength in the regression weights; with a medium relationship strength, it was associated with MD (*p* < 0.001; r = −0.344), and with a low strength, it was associated with PA (*p* < 0.01; r = −0.282). Conversely, PWP was shown to be positively related to BP (*p* < 0.001; r = 0.424), MD (*p* < 0.001; r = 0.318) and age (*p* < 0.001; r = 0.395), showing a medium-strength regression effect. Finally, a direct relationship of MD with BP (*p* < 0.05; r = 0.228) was shown, determined by a low-strength regression weight.

For females, the model fit was adequate, although this condition was not fulfilled for all indices. Chi-square analysis showed a significant p-value (X2 = 6.596; df = 1; pl = 0.010). However, the comparative fit index (CFI) obtained a value of 0.879 and the Tucker–Lewis index (TLI) obtained a value of 0.827, indicating that these parameters did not fit. However, the normalized fit index (NFI) analysis obtained a value of 0.903 and the incremental fit index (IFI) was 0.909, representing an acceptable fit. The root mean squared error of approximation analysis (RMSEA) obtained a value of 0.126. These results show that the female model did not fit as acceptably as the male model.

**Figure 5:**
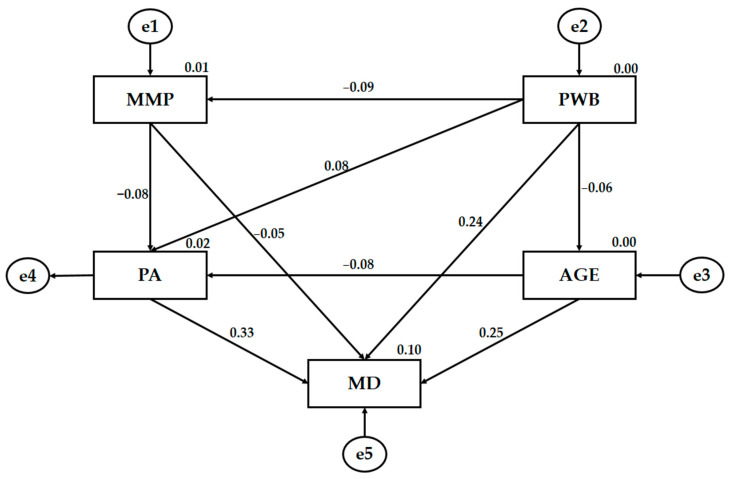
The structural equation for females. Note: Mass media pressure (MMP); Mediterranean diet (MD); physical activity (PA); psychological well-being (PWB); age (AGE); error (e).

Nevertheless, as the fit values were close to acceptable values, some statistically significant relationships were obtained. Figure 5 and Table 3 show the regression weights for the female model. In this case, the most influential variable was MD, as it was positively related to BP (*p* < 0.001; r = 0.331), determined by a medium regression weight strength, and it was also associated with PWB (*p* < 0.01; r = 0.253) and age (*p* < 0.01; r = 0.245), showing a low-strength regression effect. For the other relationships proposed in the model, no statistically significant differences were observed.

## 4. Discussion

The current study carried out among older adults addressed aspects of special relevance today, such as psychological well-being and mass media pressure, as well as their relationship with factors associated with a healthy physical education, such as the level of physical activity and the Mediterranean diet. The principal aim was to establish a structural model to explain the associations between the above variables, as well as to carry out a multi-group analysis to identify relationships according to participants’ genders. Some research with similar aspects or features has been carried out by Afshin et al. [38], Peter and Brosius, [39], Cranmer et al. [40] and Troncone et al. [41].

At present, there are few studies providing answers to the associations proposed in this research. Therefore, this study’s novelty is to provide information about the effect of mass media pressure and psychological well-being on the physical–healthy habits of people.

Mass media are a valuable tool for organizing and informing citizens; however, they frequently place a direct pressure on viewers [42,43]. The main results of this model show that the pressure applied by the mass media was negatively associated with psychological well-being, the practice of physical activity and adherence to the Mediterranean diet. Similar data are found in the study of Mieziene et al. [44], who found that mass media pressure generates a beauty ideal that seeks extreme thinness, which leads to greater personal dissatisfaction and less physical activity. Likewise, Vilhjalmsson et al. [45] argue that dissatisfaction provoked by mass media does not act as a motivating factor towards acquiring healthy habits.

Based on the above, Griffiths et al. [46] show that mass media pressure is greater on younger adults who show less psychological wellbeing. As a consequence, there is a worsening of their eating behaviour, especially based on restriction, which can lead to eating disorders.

However, findings showed that higher psychological well-being leads to an increase in healthy parameters such as physical activity and adherence to a Mediterranean diet. Sofija et al. [47] showed that wellbeing, which is manifested as a positive self-reported experience, promotes personal self-care and keeps individuals away from risky behaviours, favouring the development of behaviours focused on the protection and promotion of health [48]. Finally, an important factor, age, was included in the general model presented. In this case, it was shown that this variable had a direct impact on adherence to the Mediterranean diet. This fact can be justified and related to the fact that the adult population establishes food preferences and their consumption improves as a result of greater exposure to different food varieties [29,49].

In terms of gender differences, it should be noted that the results for men showed that mass media pressure has a negative impact on psychological well-being, leading to a decrease in physical activity and a worsening diet. According to the results obtained in the study by Izydorczyk et al. [50], the mass media pressure for men to conform to an ideal standard is evident, leading them to suffer great emotional dissatisfaction if their body size does not coincide with society’s ideal. Similarly, Tylka [51] notes that mass media pressure acts as a promoter of physical activity among men, who use it to improve their muscle tone, causing an imbalance in their eating behaviour. Likewise, Van Hecke et al. [52] and Pfister and Sisjord [53] found that mass media disseminate physical–sports activities of interest to men, which may promote physical exercise in this population sector.

However, males showed a positive personal satisfaction associated with healthy physical activity habits. These data are similar to those found by Zhang et al. [54], who found that men’s physical activity is associated with higher levels of happiness, mood and vitality, highlighting that physical exercise increases their levels of self-efficacy, which is a key factor in people’s life satisfaction [55]. Similarly, in the study by Grao-Cruces et al. [56], it is shown that physically active men show high levels of emotional well-being, as well as a positive self-concept, which favors a healthy diet that provides them with improved well-being. In addition, psychological well-being was positively influenced by age. In particular, men have been shown to perform better than women in terms of self-acceptance, purpose in life, autonomy and mastery of the environment [57]. In fact, authors such as Steptoe et al. [58] show a close relationship between wellbeing and health, as well as the increasing importance of maintaining well-being in older age.

Regarding women, it should be noted that no significant relationships were found between mass media pressure and psychological well-being and healthy habits. However, the study by Jiménez-Boraita et al. [59] shows that the mass media have a greater impact on women, deteriorating their emotional well-being.

Nevertheless, it was found that higher levels of well-being in women are associated with better healthy habits. Women showing higher levels of well-being were reported to have higher levels of healthy physical activity, as well as better eating behaviours. González-Valero et al. [60] noted that women have an extrinsic motivation to practice physical activity, as they have a specific purpose in mind; i.e., improving their physical condition, slimming their body shape, reducing their fat levels, among others. Physical exercise reduces stress levels, increases energy and improves emotional coping and control, with positive repercussions on the acquisition of healthy eating habits, as well as improved psychological well-being [61,62]. Furthermore, age was positively associated with the Mediterranean diet in this group. In other words, those individuals who were older had a better adherence to the Mediterranean diet. In fact, the study by Álvarez-Fernández et al. [63] showed that the consumption of healthy foods in a group of young women was significantly lower than in older women.

Taking the above into account, it should be noted that this is a new study, showing relationships between mass media pressure, psychological wellbeing and the acquisition of healthy habits. This enables a series of associations to be established that allow society to observe an underlying reality, as well as to verify the importance of a physically healthy education over lifetime. However, this study was not exempt from limitations, including the descriptive and cross-sectional nature of the study, which only allowed us to analyze variables at the time when they were extracted. Furthermore, due to the number of participants, the results cannot be generalized to the whole population.

Based on the results and practical applications, as future perspectives, it is intended to develop interventions that have an impact on physically healthy education from an early age, with the aim of developing emotionally stable people who do not perceive the mass media as a pressure they have to respond to, but rather as a way of promoting healthy behaviours.

## 5. Conclusions

Overall, an acceptable value was obtained for all parameters of the general equation model. This work shows that mass media have a direct influence on the well-being of the society, having a negative impact on the practice of physical activity and adherence to the Mediterranean diet. Psychological well-being is reduced as a result of these factors, leading individuals to develop eating disorders, obesity, sedentary habits or personal dissatisfaction.

Regarding the gender model, males obtained a better fit in the indices than females, although this was also acceptable. For males, it was shown that mass media pressure leads to lower emotional well-being, as well as physical activity and adherence to the Mediterranean diet. However, a better adjustment of psychological well-being was related to higher levels of regular physical exercise and better adherence to the Mediterranean diet.

As for women, no relationship was found between mass media pressure and psychological well-being and physically healthy habits. However, women showed greater personal satisfaction and reported more physical activity, as well as better eating patterns.

Therefore, in general terms, it can be concluded that the mass media have a direct influence on people’s psychological well-being. Thus, an inadequate use of mass media has a negative influence on the practice of physical activity and adherence to the Mediterranean diet. Furthermore, age will be a key factor in the development of healthy habits and the well-being of society. Therefore, the correct use of new technologies should be encouraged from an early age and special attention should be paid to physical activity and dietary recommendations in order to avoid physical and mental health problems.

## Figures and Tables

**Table 1:** The structural model for the theoretical model.

Associations between Variables			R.W.				S.R.W.
			Estimations	S.E.	C.R.	*p*	Estimations
MMP	←	PWB	−0.541	0.074	−7.324	***	−0.379
AGE	←	PWB	1.022	0.545	1.875	0.061	0.074
PA	←	MMP	−20.200	4.635	−4.358	***	−0.272
PA	←	PWB	45.742	9.001	5.082	***	0.301
PA	←	AGE	−1.103	0.628	−1.756	0.079	−0.067
MD	←	AGE	0.028	0.009	3.072	**	0.213
MD	←	PA	0.003	0.001	5.273	***	0.302
MD	←	MMP	−0.241	0.069	−3.493	***	−0.235
MD	←	PWB	0.680	0.135	5.043	***	0.296

Note 1: Regression weights (R.W.); standardized regression weights (S.R.W.); estimation error (S.E.); critical ratio (C.R.). Note 2: Mass media pressure (MMP); Mediterranean diet (MD); physical activity (PA); psychological well-being (PWB); age (AGE). Association between variables (←). Note 3: *p* < 0.01 (**); *p* < 0.001 (***).

**Table 2:** The structural model for males.

Associations between Variables			R.W.				S.R.W.
			Estimations	S.E.	C.R.	*p*	Estimations
MMP	←	PWB	−0.851	0.084	−10.141	***	−0.618
AGE	←	PWB	3.394	0.655	5.180	***	0.395
PA	←	MMP	−22.833	7.853	−2.908	**	−0.282
PA	←	PWB	66.676	13.351	4.994	***	0.424
PA	←	AGE	−0.709	1.005	−0.705	0.481	−0.040
MD	←	AGE	0.018	0.017	1.037	0.300	0.057
MD	←	PA	0.002	0.001	2.212	*	0.228
MD	←	MMP	−0.530	0.134	−3.956	***	−0.344
MD	←	PWB	0.777	0.234	3.322	***	0.318

Note 1: Regression weights (R.W.); standardized regression weights (S.R.W.); estimation error (S.E.); critical ratio (C.R.). Note 2: Mass media pressure (MMP); Mediterranean diet (MD); physical activity (PA); psychological well-being (PWB); age (AGE). Association between variables (←). Note 3: *p* < 0.05 (*); *p* < 0.01 (**); *p* < 0.001 (***).

**Table 3:** The structural model for females.

Associations between Variables			R.W.				S.R.W.
			Estimations	S.E.	C.R.	*p*	Estimations
MMP	←	PWB	−0.178	0.110	−1.616	0.106	−0.086
AGE	←	PWB	−0.980	0.826	−1.186	0.235	−0.063
PA	←	MMP	−9.286	6.146	−1.511	0.131	−0.080
PA	←	PWB	20.272	12.743	1.591	0.112	0.084
PA	←	AGE	−1.169	0.818	−1.428	0.153	−0.076
MD	←	AGE	0.034	0.011	3.018	**	0.253
MD	←	PA	0.003	0.001	4.524	***	0.331
MD	←	MMP	−0.085	0.173	−1.018	0.309	−0.052
MD	←	PWB	0.492	0.234	2.842	**	0.245

Note 1: Regression weights (R.W.); standardized regression weights (S.R.W.); estimation error (S.E.); critical ratio (C.R.). Note 2: Mass media pressure (MMP); Mediterranean diet (MD); physical activity (PA); psychological well-being (PWB); age (AGE). Association between variables (←). Note 3: *p* < 0.05 (*); *p* < 0.01 (**); *p* < 0.001 (***).
